# The Urban Heat Island in an Urban Context: A Case Study of Mashhad, Iran

**DOI:** 10.3390/ijerph16030313

**Published:** 2019-01-24

**Authors:** Marzie Naserikia, Elyas Asadi Shamsabadi, Mojtaba Rafieian, Walter Leal Filho

**Affiliations:** 1 Department of Urban Planning, Tarbiat Modares University, Nasr, Jalal AleAhmad, Tehran 14115-335, Iran; m.naserikia@modares.ac.ir; 2 Department of Civil Engineering, Ferdowsi University of Mashhad, Azadi Square, Mashhad 9177948974, Iran; e.asadi@mail.um.ac.ir; 3 Department of Urban Planning, Faculty of Arts and Architecture, Tarbiat Modares University, Nasr, Jalal AleAhmad, P.O.Box 14115-111, Tehran, Iran; rafiei_m@modares.ac.ir; 4 Research and Transfer Centre “Sustainable development and Climate Change Management”, Hamburg University of Applied Sciences, Ulmenliet 20, D-21033 Hamburg, Germany

**Keywords:** urban heat island, climate change, arid and semi-arid regions, land cover

## Abstract

In this study, the spatio-temporal changes of urban heat island (UHI) in a mega city located in a semi-arid region and the relationships with normalized difference vegetation index (NDVI) and normalized difference built-up index (NDBI) are appraised using Landsat TM/OLI images with the help of ENVI and ArcGIS software. The results reveal that the relationships between NDBI, NDVI and land surface temperature (LST) varied by year in the study area and they are not suitable indices to study the land surface temperature in arid and semi-arid regions. The study also highlights the importance of weather conditions when appraising the relationship of these indices with land surface temperature. Overall, it can be concluded that LST in arid and steppe regions is most influenced by barren soil. As a result, built-up areas surrounded by soil or bituminous asphalt experience higher land surface temperatures compared to densely built-up areas. Therefore, apart from setting-up more green areas, an effective way to reduce the intensity of UHI in these regions is to develop the use of cool and smart pavements. The experiences from this paper may be of use to cities, many of which are struggling to adapt to a changing climate.

## 1. Introduction

Rapid urbanization has resulted in a tremendous rise in population and man-made structures of cities. The population growth and constructed urban structures and surfaces have triggered many environmental problems [1]. Among climatological effects of human activities on the environment, urban heat island (UHI) is a well-documented phenomenon [2]. 

Wamsler et al. [3], in 2013, outlined the links between planning and climate change in urban areas and drew attention to such a problem. The UHI is a phenomenon where local air temperatures of urban environments are higher than the suburban rural areas. Absorption of solar radiations by mass building structures, roads, and other hard surfaces during the daytime is considered as the primary cause of heat island in densely built urban areas [1]. In addition, changes in land use/land cover (LULC) of cities can raise the temperature of the local air and surfaces several degrees higher than the temperatures of the surrounding areas at the same time [4,5]. The interplay between human activities and the environment is known as LULC and considerably influences the urban climate [6]. Various LULC types, properties of surface materials such as albedo and emissivity, air movement, anthropogenic heating, air pollution, and urban geometry are responsible for forming different UHI patterns [7,8,9,10]. Moreover, there are several LULC parameters affecting UHIs including the abundance of green biomass, vegetation cover, barren land, water, low/medium/high-density built-up areas, impervious surfaces, vegetation abundance, and impervious surface areas, of which the last two are considered the most important factors. Nevertheless, the correlation between these variables and land surface temperature (LST) is different in various studies, because the measurement of variables and units of analyses vary from study to study [11]. 

Generally, UHI studies with observational data are divided into two categories: (a) Investigating the UHI through measuring the air temperature, by running transects, and weather station data and (b) measuring LST through the use of remote sensing data [4]. Although using in situ data has the advantage of a high temporal resolution and recorded data over a long period, the poor spatial resolution is the problem. In contrast, remote sensing thermal images have the desirable spatial coverage and enable investigation of the urban canopy layer (UCL) heat island. Remote sensing data are effective measures to monitor the environment of cities particularly understanding the LULC changes, and rapid urban growth and to estimate UHI properties at the land surface level [6]. In the recent decade, advances in atmospheric sensing and improvements of sensors’ spatial and temporal resolutions have improved measuring and investigating UHIs and urban climates. A variety of sensors and platforms have been used to make thermal images of the LSTs over a range of scales in order to study the roots of UHI in urban environments [12]. 

Primary investigations of the UHI effects through remotely sensed data were conducted using the polar-orbiting Advanced Very High Resolution Radiometer (AVHRR) thermal scanner to produce one-kilometer resolution surface temperature patterns [13], to compute vegetation index and radiative surface temperatures [14], and to determine albedos and equivalent blackbody temperatures [15]. Higher resolution thermal data were rarely used to obtain LST until the 1990s. In 1990, Carnahan and Larson [16] used Thematic Mapper (TM, 120 m) data to assess mesoscale temperature differences between urban and surrounding areas in both qualitative and quantitative terms in central Indiana, including the Indianapolis metropolitan area, whereas Nichol [17] used it to indicate microclimate temperature differences in high-rise housing estates of Singapore, in 1994. However, converting the digital number (DN) into radiant temperatures, Weng [18], in 2001, derived surface radiant temperatures from radiometrically corrected TM thermal infrared data (band 6) in order to measure the changes in surface temperature in an 8-year period. Several algorithms have been recently developed for the purpose of retrieving LST from Landsat data, including a mono-window algorithm, a single channel algorithm, image-based methods, and GIS-based spatial interpolation methods [6]. Time series thermal data obtained from satellites can be effectively used to evaluate the pattern of UHI related to LULC changes [19] particularly during the daytime and warm period, the best time for observing surface UHI [20]. Many researchers have studied UHIs in different cities around the world, and the relationship between LULC and the pattern, and the intensity of UHI, some of which are reviewed in Table 1.

A large number of studies have investigated urban heat island over megacities around the world, indicating the increasing trend of the UHI intensity and expansion. The results show that the influence of the LULC on the UHI intensity, which varies depending on the location’s characteristics, is undeniable. Mashhad, which is a metropolitan city, suffers from health and environmental problems caused by anthropogenic heat produced in the last decade due to urban population growth resulting in considerable land use changes. Accordingly, the purpose of this study is to first picture the UHI in this mega city and then quantitatively assess the efficiency of the remote sensing indices related to land use and land cover patterns, namely normalized difference vegetation index (NDBI) (NDVI) and normalized difference built-up index (NDBI), in this arid region. 

## 2. Study Area

The study area is a 1400 km^2^ region in the north-east of Iran, including Mashhad metropolitan and its surrounding areas. This city is located at 36.20° North latitude and 59.35° East longitude. It is the capital of Razavi Khorasan Province and is the second most populous city in Iran, with an estimated population of about 3 million inhabitants [34], encompassing a total area of approximately 350 km^2^. Mashhad experienced rapid population growth in the last three decades. According to the censuses reported by Statistical Centre of Iran [35], its population doubled from 1987 (1,463,508 people) to 2017 (3,001,184 people). This is also a tourist city, and more than 20 million tourists visit this city from all corners of the world in summers every year [36], resulting in air pollution and excessive heat releasing during holidays.

**Figure 1:**
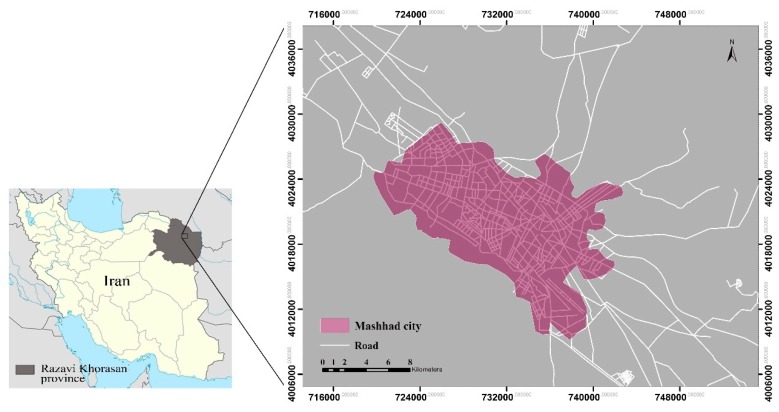
Location map of the study area.

Mashhad has a steppe climate with cool winters and hot summers. This city receives annual precipitation of 250 mm (9.8 inches) on average [37]. Summers have high temperatures which sometimes exceed 35 °C [38]. Figure 1 shows the location of the study area.

## 3. Methodology

### 3.1. Remote Sensing Data, Image Pre-Processing and LST Calculation

To examine the anthropogenic effects on the UHI in Mashhad, Iran, land cover changes and LSTs were detected between 1988 and 2017 using remotely sensed data from Landsat TM/OLI. Details of the images are described in Table 2. The images were chosen from the hottest periods of three years with the help of the Weather Underground database (http://www.wunderground.com) and (http://www.irimo.ir). The satellite images were selected considering the similarity of the time intervals, weather conditions, and minimal cloud cover. The Landsat data archive was obtained and accessed via EarthExplorer website at http://earthexplorer.usgs.gov.

FLAASH (Fast Line of sight Atmospheric Analysis of Spectral Hypercubes.) module was adopted to correct atmospheric errors, in ENVI5.3 software, after using a radiometric correction tool producing an input file (Radiance) for the FLAASH correction. The MODTRN4 radiometric transmission model, one of the most accurate atmospheric radiometric algorithms [39], is used in this model. The main variables in the module, such as central position, sensor altitude, imaging time and resolution which are available in the header file of images, have to be given for atmospheric correction [39]. Finally, land surface temperature maps were provided in ENVI5.3 software using Equation (1) [40], Equation (2) [41], Equation (3) [42], and Equation (4) [43] for the years 1988, 2001 and 2017.

(1) $$LST\left({}^{\circ}C\right)=\frac{\left(T_{b}-273.15\right)}{1+\left(\lambda \times \left(\frac{\left(T_{b}-273.15\right)}{14380}\right)\right)Ln\varepsilon },$$

(2) $$T_{b}=\frac{K_{2}}{\left\{Ln\left[\frac{K_{1}}{L_{\lambda }}+1\right]\right\}},$$

(3) $$\varepsilon =0.004\times \rho_{v}+0.986,$$

(4) $$\rho_{v}={\left(\frac{NDVI-NDVI_{min}}{NDVI_{max}-NDVI_{min}}\right)}^{2},$$

where *T_b_* (blackbody temperature) is the effective at-satellite temperature in Kelvin; *λ* is the wavelength of emitted radiance; *ε* is emissivity; *K*_2_ and *K*_1_ are pre-launch calibration constants in Wm−2 and Kelvin; Lλ is the spectral radiance in Wm−2sr−1mm−1; ρ = h × c/σ (σ is the Boltzmann constant, h is the Planck constant, and c is the velocity of light); and *ρ_v_* is the fractional vegetation.

### 3.2. Land Cover Classification

Remotely sensed data are widely used to provide land use/cover maps. Images of the years 1988, 2001 and 2017 were classified using the maximum likelihood model in ENVI5.3 to obtain land cover distribution. False color band combinations of bands (RGB = 543 for Landsat 8, RGB = 432 for Landsat 5) were utilized to help develop training samples for each LC type since these combinations provide better-categorized visualizations of urban environments. The land cover in this study area was classified into three categories, namely built-up surfaces, soil and vegetation cover. Then, confusion Matrix Using Ground Truth ROIs in ENVI5.3 was used to assess the accuracy of the maximum likelihood classification in this study and the Kappa coefficient was also obtained for each year. All the obtained information on the overall accuracy and Kappa coefficient of the LC classification are illustrated in Table 3.

### 3.3. NDVI and NDBI

NDVI and NDBI were obtained in order to study land cover changes during a period of about 30 years, and the relationship between land cover and LST. NDVI is widely calculated using Equation (5) expressing density of vegetation cover [44].

(5) $$NDVI=\frac{\rho_{ni}-\rho_{r}}{\rho_{ni}+\rho_{r}},$$

where *ρ* and *ρ* are the respective reflectance values of near-infrared and red bands of Landsat images. The NDVI value of each pixel is a number between −1 and 1, with values greater than 0 indicating vegetation cover and higher values signifies denser green lands. By contrast, NDBI (Equation (6)) can be effectively used to show built-up features. Values of this index range from −1 to 1 just the same as NDVI, but positive numbers indicate built-up areas.

(6) $$NDBI=\frac{b_{ni}-b_{mi}}{b_{ni}+b_{mi}},$$

where *b_ni_* and *b_mi_* are the digital numbers of mid-infrared and near-infrared bands of the Landsat images, respectively.

According to Zhang et al. [44], the ranges of values of these indices may vary from study to study because of the image acquisition time, and different atmospheric conditions and precipitations.

## 4. Results and Discussion

### 4.1. Land Cover Mapping

**Figure 2:**
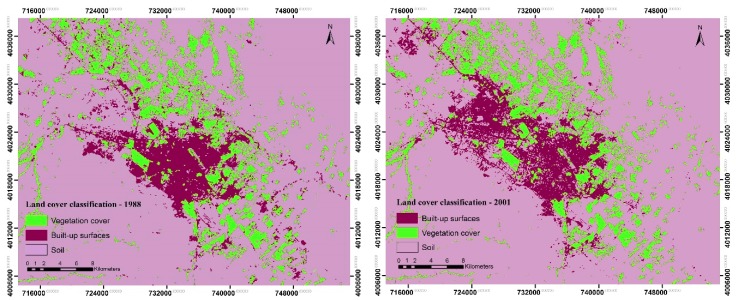

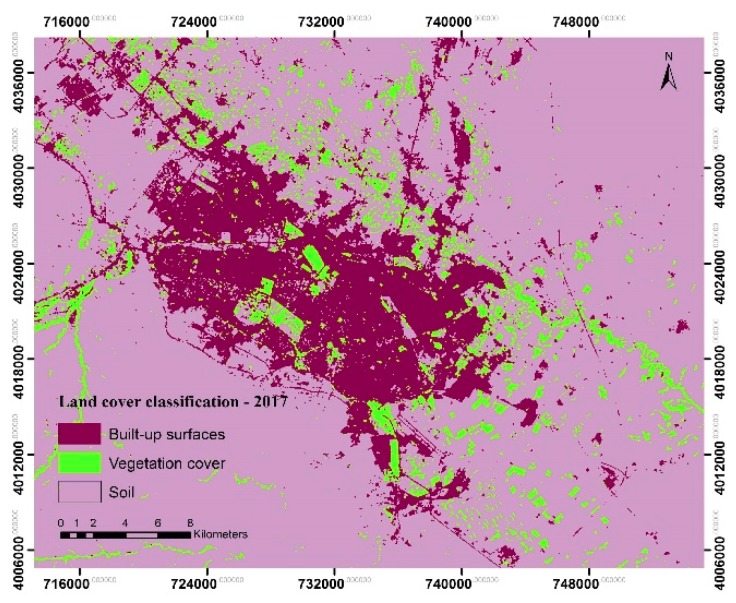
Land cover classification, 1988, 2001 and 2017.

Studying the effects of urbanization on climatic changes requires the evaluation of LC changes [5]. For this purpose, maps of the constructed areas and vegetation cover changes were produced (Figure 2). The dot distribution pattern of the constructed areas in the north-west of the city in 1988 was expanded to a chain pattern in 2001 and to a concentrated pattern in 2017, resulting in the gradual creation of irregular patterns of asphalt pavements having a considerable impact on the formation and increasing the intensity of UHI in Mashhad city during the period of about 30 years. In addition, it is worth mentioning that the concentrated urban form around the city center remained unchanged, and from in situ data, it can be said that the constructed areas in this section of the city became denser. Overall, the built-up areas expanded in different directions during the study period. 

On the other hand, the vegetation cover varied considerably from 2001 to 2017, but as can be seen in Figure 2, it remained approximately unchanged in 2001, which indicates that from 1988 to 2001 the probable development of the UHI was mainly due to the expansion of constructed areas, increase in impervious surfaces and anthropogenic heating. Moreover, the vegetation cover around the city, particularly in the north-eastern, south-eastern and northern parts, decreased in 2017, influencing the magnitude and spatial pattern of the UHI [4] in this city. Additionally, a large proportion of the mentioned areas covered with vegetation in 1988 and 2001 changed to built-up spaces in 2017. 

#### Spatial Distribution of the Constructed Areas and Vegetation Cover during the Period of Study

**Figure 3:**
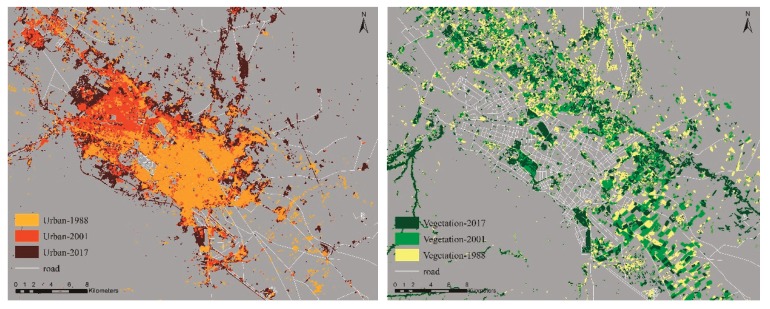
Urban land and vegetation cover in 1988, 2001 and 2017.

**Figure 4:**
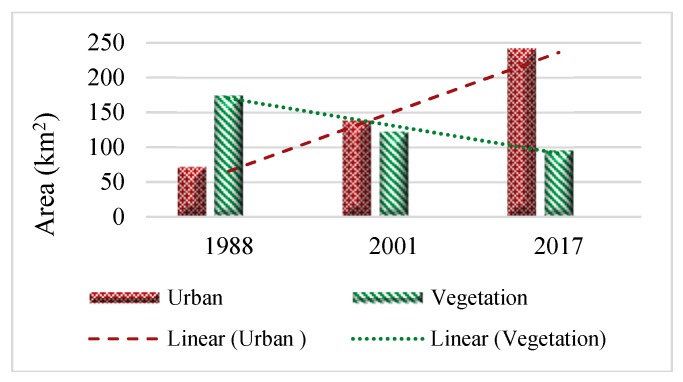
The changes of urban land and vegetation cover in the study area from 1988 to 2017.

Expansion of the built-up areas and changes in areas covered with vegetation in all the three years are shown in Figure 3, and details are presented in Table 4 and Figure 4. 

Overall, it can be seen that the constructed areas increased dramatically, while vegetation cover experienced a considerable decrease particularly from 2001 to 2017. The percentage of the built-up area increased from 5.11% in 1988 to 9.85% in 2001 and 17.29% in 2017, while the percentage of vegetation cover was 12.45%, 8.71%, and 6.80% in 1988, 2001, and 2017, respectively, with a decreasing trend. The process of urbanization in the city resulted in degradation of vegetation cover which caused aggravated UHI intensity. However, drought has forced many farmers to leave their croplands which resulted in an obvious decrease in the proportion of farmlands. Most of the farmlands turned to soil land cover in 2017, especially farmlands in the north and south-west of the study area. According to Chen et al. [5], there is a negative correlation between barren soil and temperature indicating that the turning trend of vegetation cover to bare soil definitely increases the magnitude and extent of UHI.

Mashhad experienced fast growth along with a fast change in land cover from 1988 to 2017. It is the second largest city of Iran and a large number of people traveled or immigrated to this city for different purposes, which resulted in a dramatic increase in built-up areas and infrastructures, resulting in a considerable area of impervious surfaces, contributing to UHI, to meet the needs of the increasing population. It can be said that the main contributors of the UHI in the study area were increased built-up surfaces, decreased vegetation cover, altered vegetation surfaces to barren soil, and growing population density.

### 4.2. Spatial and Temporal Distribution of LST

**Figure 5:**
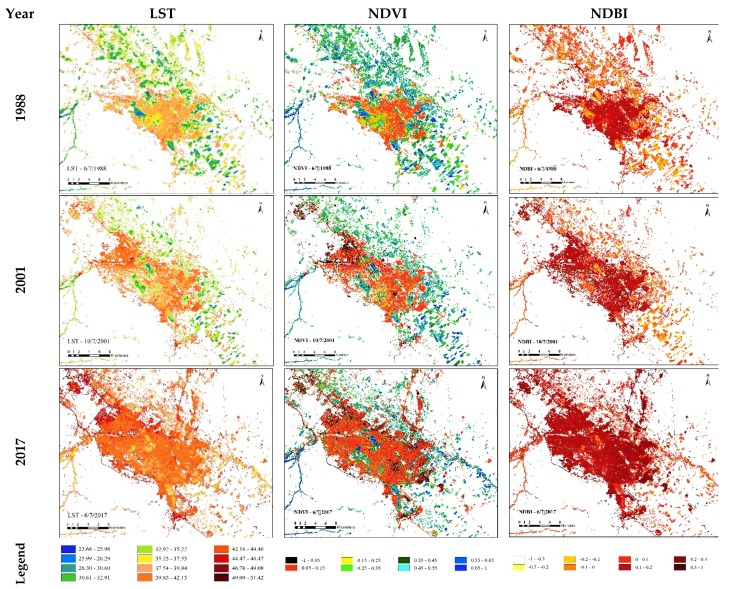
Land surface temperature (LST), normalized deference vegetation index (NDVI) and normalized difference built-up index (NDBI) in 1988, 2001 and 2017.

Since there is a causal relationship between land surface temperature and urban heat island, to further a better understanding of the distribution of the UHI clusters and changes during the two time periods, three maps for the retrieved LST of the study area were produced. The data validation was based on the accuracy of the data collection, which was checked against previous research and studies on the topic. Figure 5 indicates the temporal and spatial distribution of LST classes for 1988, 2001 and 2017. 

As seen in Figure 5, land surface temperature in the study area saw an overall increase during the period, which means that higher temperatures were observed in 2017 than 2001 and 1988. The scattered spatial pattern of the areas with relatively high temperatures in 1988 turned to a relatively contiguous one in 2001, and to a more contiguous one in 2017, along with the expansion of the regional urban system. By comparing land cover classification with LST maps, the centers of high-temperature areas were consistent with constructed features, while cool temperature surfaces were consistent with vegetation cover, particularly in 2017. It is presented in Figure 5 that growth of the UHI intensity and high temperatures in the north and north-west of the study area was affected by the urban development and replacing natural vegetation cover in these regions. Therefore, the more urbanized areas were, the more intensified UHI could be, as a result of reducing natural lands in the study area.

### 4.3. Relationship between LST and LC Types

In this part of the study, analyses of the relationships between land surface temperature and two indices, NDVI and NDBI, were used for evaluating the impact of LC types on urban thermal features.

#### 4.3.1. Relationship of LST-NDVI and LST-NDBI

To better understand the relationship between LST and NDVI, and NDBI in the study area, these indicators were mapped in Figure 5. According to the figure, temperature distribution changed in the period shown with the change of NDVI and NDBI values in the study area. In fact, the higher temperatures were found in the areas with higher NDBI and lower NDVI values, whereas lower temperatures were observed in the areas with lower NDBI and higher NDVI values. Therefore, an increase in density of built-up surfaces combined with a decrease in the vegetation density led to a noticeable rise in the temperature and UHI intensity over the past 30 years.

To quantitatively assess the urban thermal pattern, minimum, average and maximum values of the three indices (LST, NDVI, and NDBI) in 1988, 2001 and 2017 were calculated and shown in Table 5. As can be seen in Table 5, in the case of 1988, NDVI ranged from −0.024 to 0.864, while this index varied from −0.144 to 0.849 in 2001 and from −0.884 to 0.964 in 2017. Although the range of NDVI values did not considerably change, especially from 1988 to 2001, mean NDVI values experienced a downward trend from 0.322 in 1988 to 0.244 in 2001 and 0.219 in 2017. By contrast, NDBI values saw a reverse trend at the same time.

#### 4.3.2. Correlation between LST, NDVI, and NDBI

**Figure 6:**
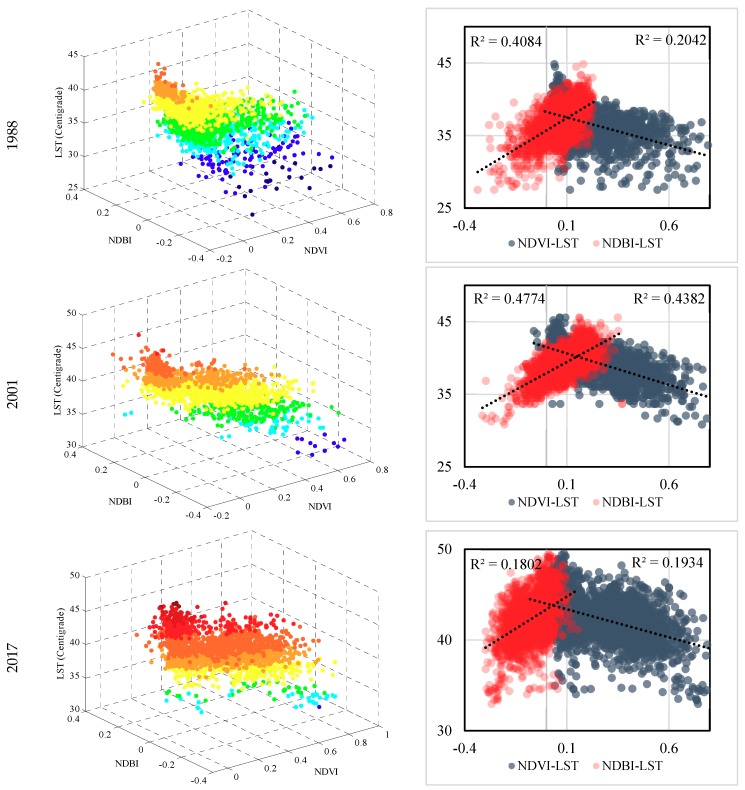
2D and 3D scatterplots of the NDBI-LST and NDVI-LST relationships.

Linear regression has been commonly used for studying the correlation of LST with NDBI and NDVI [25,33,45,46] resulting in useful equations. In this study, multiple linear regression was used to model the relationship between LST (dependent variable), NDBI and NDVI (independent variables) and data used in the regression model are pictured in a graphical form with a 3D space in Figure 6. The LST range in the 3D scatterplot was divided into 10 categories which are shown in different colors described in the figure. The top of the scatterplot is composed of pixels with high LSTs, low vegetation, and high built-up features, while the root illustrates low LST, low built-up, but highly-vegetated pixels. Between 1988 and 2017, the red colors, which represent the hotter spots, dramatically increased, particularly from 2001 to 2017. In 1988, the number of pixels with relatively cool surface temperatures was considerably greater than the ones in 2001, and the ones in 2001 showed greater cool spots than 2017. Most of the randomly selected pixels in 2017 were in the range of 37 to 52 °C indicating the increasing trend of the surface temperature. However, details of the regression model are described in Table 6, with the highest correlation in the year 2001 (0.483) and the lowest in 2017 (0.274).

On the other hand, the study suggests that surface temperatures which increased during the study period may be associated with the trend of global warming. Nevertheless, the hottest pixels in all the three years belonged to areas with NDBI that ranged between −0.05 to 0.25 and NDVI that ranged between 0 to 0.2 showing mostly built-up areas [5]. That is to say, regardless of the impact of global warming on the increasing temperature of cities, urbanization can intensify the surface temperature of different land cover types since the materials which are commonly used to build cities present low reflectivity, thermal, and optical performances [47].

The individual relationships of LST with NDVI and NDBI are illustrated in Figure 6 and Table 6. The correlation coefficients varied in all the three years with the highest correlation in 2001 and the lowest correlation in 2017, presenting that the ranges of NDVI and NDBI for specific land covers can simply change depending on many factors including the acquisition time and season [48].

**Figure 7:**
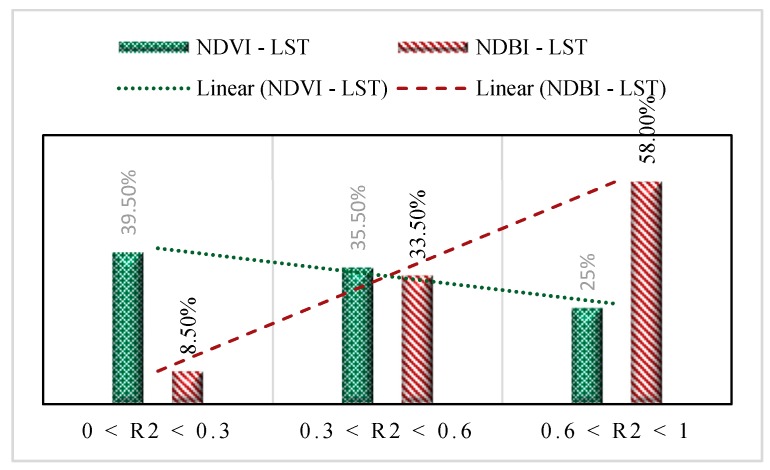
Quantitative classification of the previous works studying NDVI-LST and NDBI-LST relationships.

Overall, the correlation coefficients for all the three years were lower than 0.5 showing that these indices are not related considerably compared to the results of a number of previous studies [5,49,50,51]. The weak correlation may be associated with the climate class of the study area (arid-B). There are many random factors influencing the relationship between these indices and LST or air temperature, of which the climate conditions have a significant effect. Table 7 and Figure 7 show details of a number of studies with their Köppen-Geiger climate classes [52] (Table 8). Areas with moderate (C) and tropical (A) climates resulted in a stronger relationship between LST and the two indices, while cities located in arid (B) and cold (D) classes showed weaker correlation coefficients, mostly lower than 0.5. This result was also shown in the work done by Grover and Singh [53]. Their results showed that the LST-NDVI relationship is stronger in Mumbai (Am) than in Delhi (BSh).

There are also exceptions in each group which may be related to the land cover and the elevation of the study area, or the climate of the surroundings; in other words, there are some regions which are mostly of one specific climate class, but they are very close to other classes, for example, Khutag-Undur, which has an arid and steppe climate, is surrounded by cold regions, or Shenyang which is in a region with cold climate, dry winters, and hot summers, but is near to regions with arid and steppe, and cold with dry winters and warm summers [52]. Accordingly, the surrounding areas could have a remarkable impact on LST and air temperature of a specific area and, as a result, on the relationships of temperature with NDVI and NDBI. Mathew et al. [54] expressed that the LST-NDBI relationship in monsoon seasons is stronger than in summer and winter, indicating the importance of weather conditions, or in a larger scale the climate conditions, when estimating LST, vegetation and built-up indices.

However, the weak correlation at the end of the study period can be related to the higher LST. From a different point of view, this shows that the relationships of LST with NDVI and NDBI are stronger when the surface is covered with more vegetation, or when NDVI and NDBI are limited in range. Therefore, NDVI and NDBI are not appropriate indices for studying LST in cities that are surrounded by barren soil, which absorbs a large amount of solar radiations, like Mashhad. Mathew et al. [54] also illustrated the ineffectiveness of NDBI in SUHI studies since bare soils and dry vegetation covers show high spectral reflectance in SWIR band resulting in positive NDBI values for drier plants and higher NDBI values for the barren soil than built-up areas.

By quantitatively classifying the studies producing the correlation coefficient of the NDVI-LST and NDBI-LST relationships in three different ranges (0 to 0.3, 0.3 to 0.6, and 0.6 to 1), it is shown that more than 39% of the studies resulted in weak NDVI-LST relationships (0 < R^2^ ≤ 0.3), while just 25% of them showed strong correlations (0.6 < R^2^ ≤ 1). In contrast, the performance of NDBI has been better than NDVI, with around 58% of studies resulting in R^2^ values higher than 0.6, highlighting the relatively better efficiency of NDBI compared to NDVI in regions with extreme climate conditions. However, around 33% of the assessments of the NDBI-LST relationships resulted in an R^2^ value between 0.3 and 0.6, showing the need for a more accurate index in these regions. 

### 4.4. Implication for Findings

Our demonstration of tempo-spatial variations of the UHI in this city strongly suggests the need for including the UHI mitigation strategies in planning to reduce vulnerability under climate change. In fact, real changes are necessary to make the city more sustainable and resilient in the future. 

The most important adverse effects of UHI are related to rising temperatures in urban environments. Heat clusters in these areas can lead to increased energy consumption, increased pollution in air and water, and increased mortality and morbidity of residents [67]. Moreover, as it is concluded by Guhathakurta and Gober [68], the lot size and the effects of urban heat island can even influence the household water use; therefore, zoning regulators should consider the impact of the cover and size of the parking lots, particularly in Mashhad, and on larger a scale, in arid cities. In addition, areas surrounded by barren and semi-bare soils had a higher temperature than other areas. Therefore, indices related to vegetation cover and built-up surfaces cannot be suitable for analyzing UHI in similar cities. In fact, there is a need to use appropriate indices regarding the climate condition. Otherwise, there would be the probability of mistakes in studying UHI and choosing effective and potential mitigation strategies for future of arid and semi-arid regions.

The findings provide convincing information about land cover differences in temperature variations as a function of the type of target area’s cover and surroundings. That is to say, the role of the properties of built-up surfaces, such as the number of stories or population density is less influencing than the characteristics of the materials properties. Our findings prioritize the characteristics of building materials being used in pavements, facades, and building roofs, as the most influencing factors contributing to the intensity of urban heat island in arid regions. Therefore, city planners should provide regulations in a way to decrease the areas that are barren and for the construction industry to restrict the use of materials with high thermal capacity and low albedo, in the mentioned regions

## 5. Conclusions

Urban heat island increases the rates of energy consumption. It can also be attributed to a set of general health problems. In addition, where it occurs, UHI usually decreases the level of urban comfort. Mashhad is one of the largest cities in Iran with a large number of migrants who travel to this city for different purposes. As a result, the population of the city has increased dramatically in recent years, and the city has expanded in all directions, which has led the city to face environmental and health challenges, of which increased temperature of the city is of a high priority. According to the need for eliminating the adverse effects of this phenomenon in this city, the present study may provide a sound basis upon which action may be taken from city and local planners.

The study first investigates the tempo-spatial distribution of the UHI in Mashhad, which is located in an arid climate class according to the Koppen-Geiger climate classification, and the relationships of LST with NDBI and NDVI using Landsat TM/OLI sensor data. Second, it appraises the correlation of LST-NDBI and LST-NDVI relationships in previous works in order to evaluate the applicability of these remote sensing indices in studying LST in different climates. The brief statement of the results are as follows: (1) In Mashhad, the LST increased over the period of approximately 30 years, with weak LST-NDVI and LST-NDBI correlations; (2) the hottest spots were in areas with respective NDVI and NDBI that ranged from −0.05 to 0.25 and from 0 to 2; (3) the hottest spots were located in suburban areas; (4) the overall built-up cover increased while the vegetation cover decreased significantly, resulting in expanded UHI all over the city in 2017, particularly along with the expansion of the city towards the north; (5) according to the comparative study, the relationships between LST or air temperature and NDVI and NDBI were strongly affected by climate conditions, such as the intensity of dryness, the humidity, and the amount of monthly precipitation; (6) areas that were covered with vegetation mostly had lower temperatures than other areas particularly built-up areas, barren and semi-bare soils, especially in regions with arid climate; (7) the correlation between NDVI, NDBI and LST in regions with intensified climatic conditions (continental, arid and semi-arid) were weaker than in regions with moderate conditions.

The study shows that bare and semi-bare soils have a major influence on the UHII in arid and semi-arid cities surrounded by them, which make the use of NDVI and NDBI ineffective in these regions. In addition, these indices, particularly NDVI, are not appropriate to be used in other regions with extreme climate conditions based on the comparative study of the results of previous works. Accordingly, the application of remote sensing indices should be categorized according to the climate classification, and further studies are required to evaluate the effectiveness of various indices or developing a new index for studying the UHI in areas with extreme climate conditions.

With respect to the need for eliminating the adverse effects of urban heat island, one of the most effective ways to reduce the UHI effects in Mashhad is using cool and smart pavements since the hottest spots were allocated to the open areas covered with common pavements. Accordingly, several evaluations need to be further focused in future studies. It should be appraised whether innovative smart and cool pavements are capable of restricting the effects of the UHI in Mashhad, or, on a larger scale, in cities that are surrounded by bare soil. Moreover, appropriate land use planning besides planning for open spaces and urban green spaces in arid and semi-arid cities needs to be taken into account by city planners.

## Figures and Tables

**Table 1:** State of the art.

No.	Year	Author(s)	Data	Case Study	Study
1	2003	Streutker [4]	NOAA AVHRR	Houston, TX	The increase in the magnitude and mean area of UHI
	Results	When assessing UHI, it should be considered a dynamic meteorological feature. The outcomes of the assessment vary depending on the method of analysis. Environmental and spatial variables (such as cloud cover and vegetation cover, respectively) play important roles in the UHI extent and intensity.			
2	2004	Weng et al. [21]	Landsat ETM+	Indianapolis City, IN, USA	The LST-vegetation abundance relationship
	Results	The spatial distributions of LST are directly proportional to the variations of NDVI and the distribution pattern of green spaces. The spatial patterns of UHIs are affected by the interplay of thermal dynamics and the temporal and spatial patterns of vegetation fraction.			
3	2005	Tran et al. [12]	TERRA/MODISLandsat ETM+	Eight Asian mega cities	The UHI effects and spatial patterns
	Results	The population density may strongly influence the intensity and spatial development of UHI.			
4	2005	Chen et al. [5]	Landsat TM/ETM+	Pearl River Delta, China	The relationship between LULC changes and UHI
	Results	Land cover type can influence the temperature variations and pattern in the UHI. Temperature can be positively related with NDBI. Remote sensing indices showing vegetation cover (NDVI), and surface moisture (NDWI) are negatively correlated with temperature in cases of limited ranges of NDVI (less than 0.6).			
5	2005	Kim and Baik [22]	Automatic Weather Stations	Seoul, Korea	Tempo-spatial UHI
	Results	Increase in cloud cover and wind speed decrease the magnitude of UHI. In Seoul, the UHI was stronger on weekdays and the nighttime than weekends and the daytime, respectively, between March 2001 and February 2002.			
6	2006	Stathopoulou and Cartalis [20]	Landsat ETM+Corine database	Major cities in Greece	Thermal environment during daytime and warm period
	Results	Specific land uses and properties (including densely built-up areas near to ports) can form the hottest spots in an urban environment.			
7	2007	Jusuf et al. [23]	Landsat ETM+	Singapore	The relationship between different LULC and UHI
	Results	The thermal condition of an urban environment can be influenced by the land use type. The respective coolest and hottest land use types are park and industrial zones in daytime, and airport and commercial zones in nighttime.			
8	2009	Li et al. [24]	Landsat TM	Shanghai, China	Quantitative evaluation of UHI
	Results	Various factors can be responsible for complex patterns of UHI. Remarkable increases both in extent and magnitude of the UHI, particularly hot surfaces, in Shanghai were observed, from 1997 to 2004.			
9	2010	Tan et al. [25]	Landsat TM/ETM+	Penang Island, Malaysia	The changes in LULC
	Results	LULC changes can result in a significant Urban Heat Island Intensity (UHII). LST was strongly correlated with NDVI in all the LULC types of the study area,			
10	2011	Peng et al. [26]	MODIS Data	Global big cities	The differences in surface UHI intensity and potentially affecting biophysical and socio-economic driving factors
	Results	The difference in albedo and nighttime light affect the pattern of nighttime Surface Urban Heat Island Intensity (SUHII). There is a negative correlation between daytime SUHII and vegetation cover. Vegetation cover and green spaces have the capability to mitigate the adverse effects of UHI.			
11	2012	Li et al. [19]	Landsat TM/ETM+	Shanghai, China	Time series of LULC maps and patterns of UHIs
	Results	Green spaces, population and road density significantly relate with LST.			
12	2012	Connors et al. [27]	ASTER	Phoenix, Arizona, USA	The effects of the spatial patterns of land covers on UHI
	Results	The relative influences of urban context variations on LST associate with land use types. The relationship between LST and LULC is inconsistent for different areas and land uses. The temperature is function of urban context configuration.			
13	2014	Zhou et al. [11]	Landsat ETM+	Gwynns Falls watershed, Maryland, USA	The relationships between LST and LULC variables in different seasons
	Results	Seasonal variations do not influence the way that LULC variables affect LST prediction. Time changes the size of the context variables influence on LST prediction, with the best conditions for predicting LST in summer. During summer and autumn, vegetation covers like tree canopy, which has the high capability of restricting UHI, are appropriate variables for predicting LST. Correlation between LST and LULC is not significantly proportional to the spatial resolution of context images.			
14	2015	Fathian et al. [28]	Landsat TM/ETM+	Urmia Lake basin, Iran	The relationship between LST and LULC
	Results	Urban context variations are the most important factors determining variations of LST.			
15	2016	Amanollahi et al. [29]	Landsat TM/ETM+	Malaysia	The effects of LULC changes on the UHI
	Results	When using remotely sensed data to study changes in LULC and LST in tropical regions, the main problem would be cloudiness. The physical features of the study area, and wind magnitude are related with the UHI effects. To appraise urban LST in tropical regions, remote sensing data-GIS integration would be effective.			
16	2017	Singh et al. [30]	Landsat TM/OLI	Lucknow City, Central India	The changes in land use and the impact on UHI
	Results	Degraded ecological evaluation index in highly built-up spaces of the study area indicated the probable occurrence of undesirable eco-environmental conditions in these spaces. Over the study area, higher and lower temperatures were observed in respective densely built-up areas and green/water areas. In this study, LST was strongly correlated with NDVI and UTFVI.			
17	2017	Tran et al. [31]	Landsat TM/ETM+/OLI	Inner city area of Hanoi, Viet Nam	The relationship between LST and vegetation, man-made features, and cropland
	Results	The relationship between LST and LULC is nonlinear. Urban context configuration affects UHI.			
18	2018	Sultana and Satyanarayana [32]	Landsat ETM+	10 major metropolitan cities of India	The relationship between LULC changes and LST
	Results	Increasing number of complex UHIs existed over the Indian cities, between 2001 and 2013. Rise in built-up/urban spaces and dry/barren lands, and fall in areas covered with vegetation and green spaces result in higher UHI magnitudes.			
19	2018	Aboelnour and Engel [33]	Landsat TM/OLI/TIRS	Greater Cairo Region, Egypt	The urban sprawl with respect to LST
	Results	Degraded green spaces, caused by rapid urbanization, may be the reason behind surface heat island and undesirable urban microclimates. LST could be calculated with the help of different emissivity models, with negligible variations.			
20	2018	Silva et al. [6]	Landsat TM/OLI	Paço do Lumiar, Brazil	The influence of vegetation cover and fragmentation on the urban environment
	Results	Degraded green spaces along with densely built-up areas with increasing number of bulky man-made structures may increase the intensity UHI and thermal fluxes.			

**Table 2:** Details of the Landsat TM/OLI images.

Date of Image	DSA *	Sensor	Flight Time (GMT)	Tma (°C)	Cloud Cover (%)
6 July 1988	188	TM	06:07:52	27.4	0.00
10 July 2001	191	TM	06:17:36	27.7	0.00
6 July 2017	187	OLI	06:36:59	29	0.00

* DSA = Day of the year; and Tma = the average monthly temperature.

**Table 3:** The overall accuracy of classified land cover.

Year	Overall Accuracy	Kappa Coefficient
1988	94%	0.88
2001	97%	0.96
2017	98%	0.87

**Table 4:** The proportion of the urban land, vegetation cover and soil in the study area.

Year	Urban (km^2^)		Vegetation (km^2^)		Soil (km^2^)	
1988	71.55	5.11%	174.36	12.45%	1153.90	82.43%
2001	138.01	9.85%	122.02	8.71%	1139.77	81.42%
2017	242.14	17.29%	95.18	6.80%	1062.48	75.90%

**Table 5:** Details of the calculation of LST, NDVI and NDBI in 1988, 2001 and 2017.

Year	LST			NDVI			NDBI		
	min	max	mean	min	max	mean	min	max	mean
1988	23.68274	46.66728	35.85415	−0.02351	0.86358	0.32202	−0.37117	0.40875	0.04971
2001	24.97311	48.84806	38.38122	−0.14442	0.84903	0.24409	−0.41739	0.51931	0.09307
2017	29.40610	51.41578	41.96666	−0.88413	0.96426	0.21902	−0.46967	0.32005	−0.05193

**Table 6:** Details of the multiple linear regression model.

Year	R	R Square	Std. Error of the Estimate	Unstandardized Coefficient	Standardized Coefficient (Beta)
1988	0.680	0.46	1.77	Constant: 36.68 NDVI: −4.25 NDBI: 14.59	NDVI: −0.25 NDBI: 0.55
2001	0.703	0.49	1.54	Constant: 39.21 NDVI: −3.38 NDBI: 10.35	NDVI: −0.26 NDBI: 0.47
2017	0.450	0.20	2.31	Constant: 43.86 NDVI: −4.00 NDBI: 6.04	NDVI: −0.29 NDBI: 0.18

**Table 7:** The correlation of NDVI-LST and NDBI-LST relationships of previous case studies in various climate classes.

No.	Study Area	Climate	Date of Study	NDVI-LST		NDBI-LST	Reference
1	Mumbai, India	Am	2010	profile (North)	R^2^ = 0.59	R^2^ = 0.63	[55]
				Profile (Central)	R^2^ = 0.32	R^2^ = 0.68	
				profile (south)	R^2^ = 0.46	R^2^ = 0.61	
				N–S Profile	R^2^ = 0.35	R^2^ = 0.30	
2	Langkawi Island, Kedah, Malaysia	Am	2002	R^2^ = 0.15		R^2^ = 0.81	[56]
			2015	R^2^ = 0.5		R^2^ = 0.84	
3	Mumbai, India	Am	2010	R^2^ = 0.36		-	[53]
	Delhi, India	BSh	2010	R^2^ = 0.06		-	
4	Bangkok Metropolitan Administration	Aw-As	2008	R^2^ = 0.41		R^2^ = 0.73	[57]
5	Surat city	As-Aw	1990	R = −0.69		R = 0.68	[51]
			2009	R = −0.86		R = 0.87	
6 (air temperature)	Sukhbaatar	BSk-Dwb	2000–2009	R^2^ = 0.79		-	[58]
	Inget Tolgoi	BSk-Dwc		R^2^ = 0.97		-	
	Khutag-Undur	BSk-Dwb-Dwc		R^2^ = 0.84		-	
	Baruun-Urt	BSk		R^2^ = 0.78		-	
	Undurkhaan	BSk		R^2^ = 0.71		-	
	Khujirt	BSk		R^2^ = 0.91		-	
	Sainshand	BWk		R^2^ = 0.03		-	
	Mandalgobi	BWk		R^2^ = 0.5		-	
	Ehiingol	BWh		R^2^ = 0		-	
	Dalanzadgad	BWk		R^2^ = 0.02		-	
7	Weigan and Kuqa river oasis, Xinjiang, China	BWk	1989	R^2^ = 0.51		-	[59]
			2011	R^2^ = 0.76		-	
8	Erbil, Iraq	Csa-BSh	2003–2014	R^2^ = 0.18		-	[60]
9	Florence	Csb	2016	R = −0.71		R = 0.71	[43]
	Naples	Csb	2016	R = −0.57		R = 0.61	
10	Pearl River Delta	Cwa	2000	R^2^ > 0.98		R^2^ > 0.98	[5]
11	shenzhen	Cwa	2009–2010	R^2^ > 0.72		R^2^ > 0.51	[50]
12	Hong Kong	Cwa	2005	R = −0.41		R = 0.71	[61]
13	Guangzhou, South China	Cwa-Cfa	2000	R^2^ = 0.05		R^2^ = 0.78	[62]
			2008	R^2^ = 0.01		R^2^ = 0.71	
14	Guangzhou, South China	Cwa, Cfa	1990	R^2^ = 0.37		R^2^ = 0.53	[63]
15	Skopje, Macedonia	Cfa	2013	R = −0.63		R = 0.67	[64]
			2017	R = −0.59		R = 0.64	
16	Upper-hill, Nairobi	Cfa	1987	R^2^ = 0.26		-	[45]
			2002	R^2^ = 0.49		-	
			2015	R^2^ = 0.48		-	
			2017	R^2^ = 0.16		-	
17	Fuzhou City	Cfa or Csc	1989	R^2^ = 0.29		R^2^ = 0.87	[44]
			2001	R^2^ = 0.07		R^2^ = 0.74	
18	Wuhan City	Cfa or Csc		R^2^ = 0.79		-	[49]
19	Shenyang, China	Dwa	2001	R = −0.07		R = 0.91	[65]
			2010	R = −0.85		R = 0.91	
20	Chicago City, USA	Dfa	2010	R = −0.34		R = 0.26	[66]
21	Seven-county Twin Cities Metropolitan Area (TCMA) of Minnesota.	Dfb	2002	R^2^ = 0.09		-	[48]
			2002	R^2^ = 0.05		-	
			2000	R^2^ = 0.02		-	
			2001	R^2^ = 0.11		-	

**Table 8:** Köppen climate classification scheme symbols description [52].

1st	2nd	3rd
A (Tropical)	f (Rainforest)	
	m (Monsoon)	
	w (Savanna, Wet)	
	s (Savanna, Dry)	
B (Arid)	W (Desert)	
	S (Steppe)	
		h (Hot)
		k (Cold)
C (Temperate)	s (Dry summer)	
	w (Dry winter)	
	f (Without dry season)	
		a (Hot summer)
		b (Warm summer)
		c (Cold summer)
D (Cold (continental))	s (Dry summer)	
	w (Dry winter)	
	f (Without dry season)	
		a (Hot summer)
		b (Warm summer)
		c (Cold summer)
		d (Very cold winter)

## Abbreviations

UHI	Urban Heat Island
UHII	Urban Heat Island Intensity
SUHII	Surface Urban Heat Island Intensity
LULC	Land Use/Land Cover
LST	Land Surface Temperature
UCL	Urban Canopy Layer
DN	Digital Number
LC	Land Cover
NDVI	Normalized Difference Vegetation Index
NDBI	Normalized Difference Built-Up Index
